# Comprehensive Assessment of a Hotspot with Persistent Bancroftian Filariasis in Coastal Sri Lanka

**DOI:** 10.4269/ajtmh.18-0169

**Published:** 2018-07-16

**Authors:** Ramakrishna U. Rao, Sandhya D. Samarasekera, Kumara C. Nagodavithana, Charles W. Goss, Manjula W. Punchihewa, Tharanga D. M. Dassanayaka, Udaya S. B. Ranasinghe, Devika Mendis, Gary J. Weil

**Affiliations:** 1 Department of Internal Medicine, Washington University School of Medicine, St. Louis, Missouri;; 2 Ministry of Health, Anti-Filariasis Campaign, Colombo, Sri Lanka;; 3 Division of Biostatistics, Washington University School of Medicine, St. Louis, Missouri;; 4 Regional Anti-Filariasis Unit, Galle, Sri Lanka

## Abstract

The Sri Lankan Anti-Filariasis campaign distributed five rounds of mass drug administration (MDA with diethylcarbamazine plus albendazole) to some 10 million people in eight districts between 2002 and 2006. Sri Lanka was recognized by the WHO for having eliminated lymphatic filariasis (LF) as a public health problem in 2016. However, recent studies by our group documented pockets with persistent LF in coastal Sri Lanka, especially in Galle district. The present study was performed to reexamine an area previously identified as a potential hotspot for persistent LF (Balapitiya Public Health Inspector area, population 17,500). A community survey documented high rates for circulating filarial antigenemia (3%, confidence interval [CI]: 1.8–4.9) and microfilaremia (1%, CI: 0.5–2.5%). Circulating filarial antigenemia rates were 2.8-fold higher in males than females. High prevalence was also observed for anti-filarial antibodies in young children (5.7%, CI: 3.7–8.4%) and for filarial DNA in vector mosquitoes (5.2%, CI: 4.2–6.3%). Spatial data showed that persistent LF was dispersed across the entire study area. Other studies showed that persistent LF was not limited to Balapitiya and not solved by additional rounds of MDA. Molecular xenomonitoring studies conducted in 2016 in 22 of 168 Public Health Midwife areas in the coastal Galle evaluation unit (approximate population 600,000) found that 179 of 660 (27%) pools of *Culex* collected from all areas were positive for *Wuchereria bancrofti* DNA by quantitative polymerase chain reaction; the estimated infection rate in mosquitoes was 1.26%, CI: 1.0–1.5%. Interventions other than routine MDA will be required to remove LF hotspots in Balapitiya and in other areas in coastal Sri Lanka.

## INTRODUCTION

Lymphatic filariasis (LF) has been endemic in Sri Lanka for hundreds of years with the highest rates in the “filariasis belt” in the western and southern parts of the country.^1–3^ The Anti-Filariasis Campaign (AFC, established in 1947) implemented a variety of control activities over many years that reduced infection prevalence to low levels by 1999. After providing mass drug administration (MDA) with diethylcarbamazine (DEC) for three years starting in 1999, the AFC provided five annual rounds of MDA with DEC plus albendazole in all eight endemic districts (implementation units, IU) between 2002 and 2006.^4–6^ The AFC conducted post-MDA surveillance activities according to the WHO guidelines, and all evaluation units (EUs) in endemic districts easily passed transmission assessment surveys (TAS) in 2013.^7^ These surveys are designed to test whether filarial antigenemia prevalence in young school children is less than 2% with 95% certainty.^8^ However, prior studies by our group have shown that TAS was not sensitive for detecting ongoing transmission of *Wuchereria bancrofti* in many areas in Sri Lanka,^9^ and this is likely to be true in other areas where LF is transmitted by *Culex* mosquitoes. This suggests that children aged 6–8 years may not be good sentinels for detecting residual infections in this type of setting but their antibody responses can be good markers for recent transmission.

Based on the encouraging TAS results and other criteria, the WHO recognized that Sri Lanka had eliminated LF as a public health problem in 2016 but recommended that the country continue surveillance efforts and intervention to clear residual infections in foci with persistent infections.^10,11^

Lymphatic filariasis is highly focal infection, and the epidemiological models suggest that the distribution of the infection may become more heterogeneous following multiple rounds of MDA.^12,13^ We performed comprehensive LF surveys in 19 Public Health Inspector (PHI) areas that were considered to be at risk for persistent infection in 2011–2013.^9^ The surveillance package included community surveys for circulating filarial antigenemia (CFA) and microfilaremia (Mf), school surveys for CFA and anti-filarial antibodies, and molecular xenomonitoring (MX, systematic sampling and testing *Culex quinquefasciatus* for filarial DNA by PCR).^9^ All 19 sentinel areas studied had evidence for persistent LF, but some areas had stronger signals than others. Based on results of that study, we suggested revised endpoint targets (upper 95% confidence intervals [CIs]) for filariasis elimination programs in areas with *Culex* transmission as follows: community CFA, < 2%; anti-filarial antibody prevalence in first and second grade primary school children, < 5%; prevalence of filarial DNA in *Culex* mosquitoes (fed, gravid, or semi-gravid, collected with gravid traps), < 1%.^9^ Follow-up surveys conducted in six PHIs that had strong signals in 2011–2013 (approximately 3 years after the previous surveys) found that two PHIs in Galle district and one in Matara district failed to meet these endpoint targets in 2015–2016.^14^ These results and a district-wide MX study in the Galle district coastal EU (population ∼600,000) suggested that there were many hotspots with persistent infection and transmission in that EU despite the fact that it had easily passed pre-TAS and TAS surveys that were conducted according to the WHO guidelines in 2013.^15^ We now report results of comprehensive surveys that were performed in a suspected LF hotspot within the Galle coastal EU, namely the Balapitiya PHI area. The study also reports results from other post-MDA surveillance activities conducted in the Galle coastal EU after 2015.

## METHODS

### Study sites.

The Balapitiya Medical Officer of Health (MOH) area is located in southwestern Sri Lanka; it is bordered by the Indian Ocean to the west and by Bentota and Ambalangoda MOH areas that also had evidence of persistent LF in recent surveys. The Balapitiya MOH area comprised five PHI areas, and these comprised 22 Public Health Midwife (PHM) areas with an average population of 3,500 (range 2,400–5,100). Much of the present study was conducted in the Balapitiya PHI area in the MOH area of the same name. Five PHM areas containing 11 Grama Niladhari divisions are within the Balapitiya PHI area.

In an attempt to mop-up pockets with persistent filariasis in Galle district, the AFC provided MDA with DEC plus albendazole in 14 MOH areas in the district (including Balapitiya) in September 2014 and in September 2015. The reported MDA compliance rates for Galle district were 72% and 85% in 2014 and 2015, respectively. Compliance rates in the Balapitiya MOH area for these years were 78% and 82% (AFC unpublished reports). This study reports results of a comprehensive LF survey in the Balapitiya PHI area that was conducted between March and June in 2015, some 6 months after the round of MDA that was provided in 2014. Additional MX surveys were also conducted between March and May in 2016 (6 months after the 2015 MDA) in eight of the 22 PHM areas in the Balapitiya MOH area, and the areas surveyed included some PHM areas that were also surveyed in 2015. The 2016 MX surveys also sampled 14 additional PHM areas in other MOH areas within the Galle coastal EU that had signals for persistent LF in prior surveys.

### Assessment of LF infection parameters in the Balapitiya PHI area.

Field procedures to assess LF infection parameters were essentially the same as those previously described.^9,14^ Briefly, field teams for collection of demographic information and blood samples comprised a medical officer, a PHI, a data entry technician, a phlebotomist, and one or two assistants. Finger-prick blood samples were collected by sterile, single-use, contact-activated BD-microtainer lancets (Fisher Scientific, Pittsburgh, PA) into an ethylenediaminetetraacetic acid (EDTA)-coated blood collection vial (Fisher Scientific) during the day from consenting participants. Preprinted linear barcode labels (Partnered Print Solutions, Atlanta, GA) were used to link samples to participant records. Samples were transported to the AFC laboratory in Colombo, in coolers for filarial antigen testing. Plasma was separated from finger-prick blood samples from school children by centrifugation and stored at −80°C for later antibody testing.

### Surveillance methods in communities and in primary schools.

Comprehensive surveys for LF parameters were performed as previously described.^9^ Balapitiya MOH and PHI area maps, census information (numbers of houses, schools, and numbers of primary grade school children) were obtained from census records, voter lists, and from school principals and administrators. Household sampling was achieved by sampling an equal number of houses from all quadrants in each of the five PHM areas within the Balapitiya PHI area with the goal of sampling approximately 140 houses and 500 people (ages 10–70).

Primary grade school children (grades 1 and 2, ages 6–8) in all five schools that serve the Balapitiya PHI area were enrolled in the study. No sampling was involved, so all children with parental consent were tested.

Immunochromatographic card test (ICT) card tests (BinaxNOW^®^ Filariasis, Alere, Inc., Scarborough, ME) were used for detecting CFA in blood samples.^16^ One hundred microliter of whole blood collected in EDTA from a finger prick was used for antigen testing according to the manufacturer’s instructions (Alere, Inc.). Card tests were read visually at 10 minutes as per the manufacturer’s instructions, and results were recorded using a tablet (Google Nexus-7; ASUS Computer International, Fremont, CA) containing the survey data entry forms.

Immunoglobulin G4 antibodies to recombinant filarial antigen Bm-14 in human plasma were detected by microplate enzyme linked immunosorbent assay (ELISA) (Filariasis CELISA, Cellabs Pty Ltd, Brookvale, Australia) as previously described.^9,17^ Plasma samples were tested in a single well per sample. All samples with positive or borderline test results (optical density [OD] values > 0.35) were retested on a different day to confirm positivity. Samples with two OD values > 0.35 were considered to be positive.

Microfilaria testing was performed for persons with positive ICT card tests with three-line blood smears (60 μL total volume) prepared with finger-prick blood collected between 9 pm and 12 midnight. Blood smears were air-dried, fixed, stained with Giemsa, and examined by microscopy to detect microfilariae.

### Anti-Filariasis Campaigns community Mf survey.

The AFC conducted a large-scale night blood Mf survey in 2016 following two rounds of MDA that were provided in 2014 and 2015. Residents aged ≥ 2 years in randomly selected households (HHs) within two public health field office areas (Balapitiya and Wathugedara) within the Balapitiya MOH area were tested. Field teams collected finger-prick blood (two blood smears with 30 μL of blood each) at night after 20:30 hours. Slides were stained with Giemsa, and Mf was detected by microscopy in the AFC central laboratory in Colombo and in the Regional Filariasis Unit laboratory in Galle.

### Mosquito collection for *W. bancrofti* DNA detection.

*Culex quinquefasciatus* were collected with Centers for Disease Control gravid traps (Model 1712, John W. Hock Company, Gainesville, FL) as previously described.^9,15^ Consent was obtained from heads of HHs to place traps next to their houses for 1–3 nights. In the Balapitiya PHI survey conducted in 2015, 50 traps were placed in this PHI for collecting *Culex*. To sample all areas within the PHI area, 10 traps were placed in each of the five PHM areas that comprise the PHI area. Traps were placed outside of houses in shaded areas in four quadrants of each PHM to systematically sample all areas in the PHI area. Four pools of 20 mosquitoes were collected from each trap in the PHI area. A total of 200 pools were collected in 50 trap sites.

For MX surveys conducted in 2016, traps were placed in 22 PHMs (eight in the Balapitiya PHI and 14 in other areas within the coastal Galle EU) that had high LF infection parameters in prior surveys. Traps were placed for 1–3 nights in each quadrant of each PHM to ensure broad sampling. Two pools of 25 mosquitoes were collected from each trap location. Mosquitoes were sorted, dried at 95°C for 1 hour, and placed in tubes for transport to the AFC laboratory for testing.

### DNA extraction and filarial DNA detection by qPCR from dried mosquitoes.

Dried mosquitoes (limited to blood-fed, gravid, and semi-gravid *Cx. quinquefasciatus*) were placed in Seal-Rite 2.0-mL round-bottomed microcentrifuge tubes (USA Scientific, Inc. Ocala, FL). These were sorted into four pools of 20 mosquitoes in each tube (2015 survey) or into two pools of 25 mosquitoes (2016 survey). Approximately, 200 pools were collected from the Balapitiya PHI area in 2015 and 660 pools from the other areas in Balapitiya MOH area and other high-risk areas in the coastal Galle EU in 2016. Isolation of DNA from mosquitoes was performed as previously described using Qiagen DNA extraction kits (Germantown, MD).^9,15^ DNA samples were stored at −20°C in sterile tubes that were labeled with barcodes. *Wuchereria bancrofti* DNA was detected in mosquito pools by qPCR as previously described.^18^

### Data collection, data management, and analysis.

Demographic information from participants was collected and entered into Motorola BLU mobile telephones (Motorola Solutions, Inc., Schaumburg, IL) using preloaded survey forms produced with LINKS data collection software (https://www.linkssystem.org). Self-reported information on the ingestion of anti-filarial medications during national MDA program in 2000–2006, bed net use while sleeping the previous night, and clinical conditions such as lymphedema and hydrocele (in males) was collected. Participant data, their specimens, and test results were linked to study identification numbers using preprinted barcode labels. De-identified, cleaned data were transferred into Microsoft Excel files (Microsoft Corp., Redmond, WA) for analysis.

### Spatial analysis.

Household locations were mapped using ArcGIS 10.5.1 (ESRI, Redlands, CA). Symbols were color coded to show the infection status of HH residents and HH trap locations for mosquito pools that were collected. ArcGIS 10.5.1 was used for spatial analysis. Household and trap locations were converted to UTM zone 44N WGS84 coordinates for proximity analysis. Euclidean distances in meters from each of the HHs to the closest positive trap were determined using the spatial join function and then tested to find whether HHs with one or more occupants with positive CFA or Mf tests were significantly closer to positive mosquito traps than negative HHs.

### Data analysis.

Fisher’s exact test and χ^2^ analyses were used to assess the significance of differences in prevalence for categorical filariasis parameters such as antigenemia and antibody in human blood samples and filarial DNA in mosquitoes. Relationships between human and mosquito infection parameters were assessed by the nonparametric Spearman rank test. Relationships between risk factors and antigenemia and Mf were assessed using a generalized estimating equations logistic regression approach, which adjusts estimates for correlation among subjects within a HH (SAS 9.4, SAS Institute Inc., Cary, NC). Graphs were produced with GraphPad Prism 7 software (La Jolla, CA). Filarial DNA prevalence (maximum likelihood estimates with 95% CIs) was calculated with PoolScreen 2.02 (University of Alabama at Birmingham, Birmingham, Alabama).^19,20^

### Ethical approval.

The study protocol for comprehensive surveillance was reviewed and approved by institutional review boards at the University of Kelaniya in Sri Lanka and at the Washington University and by the Sri Lanka Ministry of Health. Before school surveys, AFC personnel held formal meetings with officials from the Sri Lankan Ministry of Education, school principals, and groups of parents/guardians to discuss the aims and procedures for the study and to provide awareness about the significance of the study. Anti-Filariasis Campaign provided anti-filarial medications as per WHO guidelines to treat infected persons who were identified during these studies. Anti-Filariasis Campaign provided MDA to all participants in the EU that were present during MDA campaigns regardless of their infection status in prior surveys.

Printed copies of participant information sheets and written consent forms were provided to participants (or to parents/guardians) in Sinhalese, Tamil, and English. Written consent was obtained from adults. Participation of minors required written consent from at least one parent or guardian plus assent by the child/minor.

## RESULTS AND DISCUSSION

### Balapitiya community survey results.

**Figure 1.**
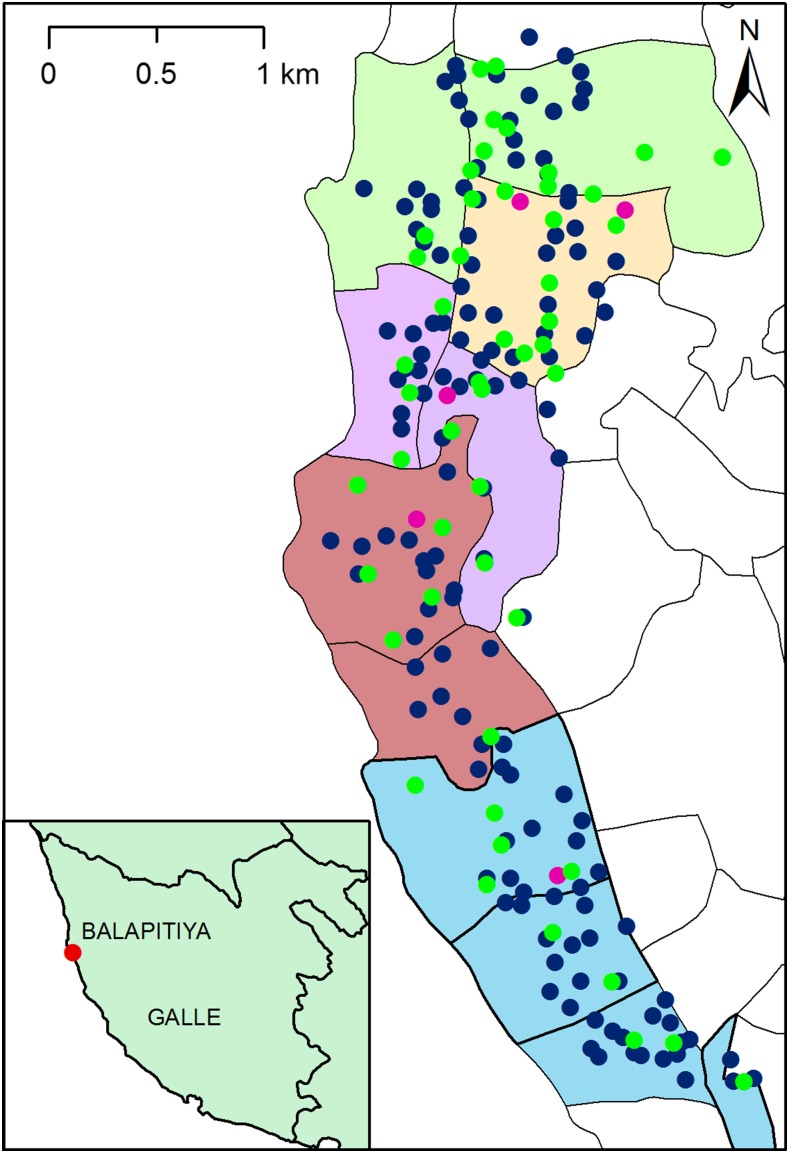
The map shows locations for households, primary schools, and mosquito collection sites tested for filarial infection in the Balapitiya Public Health Inspector (PHI) area. Blue and pink circles are locations of houses and schools selected in this area, respectively. Green circles indicate trap locations for collecting *Culex quinquefasciatus*. Five Public Health Midwife areas within the PHI are shown in different colors. The geographic lines in the map represent Grama Niladhari boundaries (Lowest administrative divisions covering Balapitiya PHI area). The map (inset) shows the location of the Balapitiya Medical Officer of Health area in Galle district. This figure appears in color at www.ajtmh.org.

The Balapitiya PHI area (comprised five PHM areas) was surveyed for LF between March and June 2015 (Figure 1). Five hundred and twenty-eight people from 140 HH were enrolled in community surveys (ages 10–70, mean age 36 years). Approximately, 37% of the population enrolled in the study was males; many participants (71.5% males and 70% females) reported swallowing MDA medications at least once during the national MDA campaign in 2002–2006. Small percentages of the surveyed population reported that they had lymphedema or hydrocele (0.8% and 0.5%, respectively). Sixty-five percentage of community survey participants reported that they had slept under a bed net during the night before their enrollment in the study.

**Figure 2.**
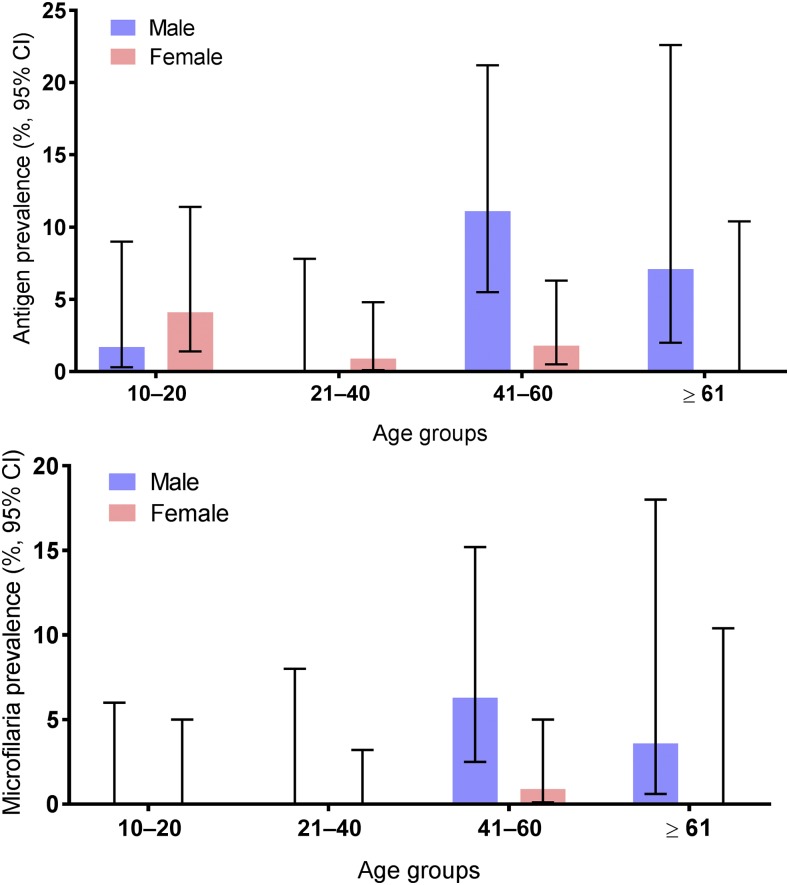
This figure illustrates filarial antigenemia and microfilaremia rates by age and sex in the Balapitiya Public Health Inspector area. These results show that although some teenagers were infected, most residual infections in the study area were in adult males older than 40 years. CI = confidence interval. This figure appears in color at www.ajtmh.org.

Table 1 shows combined survey results for *W. bancrofti* infection parameters in the PHI. Community CFA prevalence was 3.0%, and CFA positivity tended to be more common in males than females (10 of 196 males or 5.1%, CI: 2.8–9.1 versus six of 332 females or 1.8%, CI: 0.8–3.9%, *P* = 0.06). Microfilaremia also tended to be more common in males (5/196 or 2.6%, CI: 1–6% versus one of 332 or 0.3%, CI: 0.05–1.7%, *P* = 0.05) (Figure 2). Community Mf prevalence (age ≥ 10) was 1.1% (CI: 0.5–2.5%), and this was higher in adults (age ≥ 18 years) than in school age children (aged 10–17 years who participated in the community survey) (1.4%, CI: 0.6–3.2% versus 0%, CI: 0–3.7%, *P* = 0.2). Most persons with positive CFA tests and all persons with Mf were over 40 years. Six of 16 CFA-positive adults had Mf (mean Mf count 18 per 60 μL, range 2–58), and five of six Mf-positive participants were adult males.

**Table 1:** *Wuchereria bancrofti* infection parameters in community survey participants and in school surveys in five Public Health Midwife (PHM) areas within the Balapitiya Public Health Inspector (PHI) area in Galle district, Sri Lanka

			Community surveys		School surveys		
PHI	PHM	Pop. (size)	Mf prevalence positive/total (%, 95% CI)	CFA prevalence positive/total (%, 95% CI)	CFA prevalence positive/total (%, 95% CI)	Mf prevalence positive/total (%, 95% CI)	Antibody prevalence positive/total (%, 95% CI)
Balapitiya	Galmangoda	2,461	0/74 (0)	0/74 (0)	1/14 (7.1)	0/14 (0)	0/14 (0)
	Brahmanawathugoda- South	3,140	2/95 (2.1)	2/95 (2.1)	NA	NA	NA
	Balapitiya	3,381	1/103 (1)	3/103 (2.9)	2/107 (1.9)	1/107 (0.9)	7/104 (6.7)
	Randombe	5,180	2/155 (1.3)	7/155 (4.5)	2/24 (8.3)	0/24 (0)	3/21 (14.3)
	Brahmanawathugoda-North	3,363	1/101 (1)	4/101 (4)	0/256 (0)	0/256 (0)	12/248 (4.8)
	Total	17,525	6/528 (1.1, 0.5–2.5)	16/528 (3.0, 1.8–4.9)	5/401 (1.2, 0.5–2.8)	1/401 (0.2, 0–1.4)	22/387 (5.7, 3.7–8.4)

Antibody = antibody to recombinant filarial antigen Bm-14; CFA = circulating filarial antigen; CI = confidence interval; Mf = Microfilaremia; NA = no school survey was performed in this PHM; Pop. = population.

Logistic regression was used to assess several risk factors for filarial antigenemia and Mf. Male gender was a significant risk factor for antigenemia (Odds ratio 2.93 [CI: 1.05–8.2], *P* = 0.04) and for Mf (Odds ratio 8.48 [CI: 1.003–71.7, *P* = 0.05]). This male predominance is similar to that recently reported from other areas in Sri Lanka.^9,14,21^ Age, bed net use, and prior treatment of LF were not significant risk factors for CFA or Mf. This may be because this study was not adequately powered to assess the significance of these risk factors in a low prevalence area. Higher infection rates in adult males may be due to higher baseline infection rates and/or to lower MDA compliance in males.

These results from Balapitiya PHI illustrate a weakness in the current WHO guidelines for post-MDA surveillance that require pre-TAS sampling in only a few sentinel and spot check sites within large EUs to qualify for TAS. Pre-TAS targets are 1% for Mf and 2% for CFA. If the Balapitiya PHI had been one of the sentinel or spot check sites sampled in pre-TAS surveys, the coastal Galle EU would not have qualified for TAS, and it would have required additional rounds of MDA.

### School survey results.

First and second grade children in all five schools that serve the PHI were tested. Three hundred and eighty-eight blood samples were tested for CFA, and 387 plasma samples were tested for antibody to Bm-14 antigen by ELISA. Five children were CFA positive (Table 1) and one of these (a female) was Mf positive (24 Mf/60 μL). Three of the five schools surveyed had at least one child with a positive CFA test. The overall CFA prevalence in the school children was 1.2% with an upper CI value of 2.8%. Thus, a school-based TAS limited to this part of Galle district probably would have failed.

The anti-filarial antibody prevalence in the school survey (5.7%, CI: 3.7–8.4%) far exceeded the prevalence and upper confidence limit targets that we have advocated in prior publications (2% and 5%, respectively, see Table 1 and refs. 9 and 14). About half of the children surveyed were males (53%), and there was no significant gender difference in CFA or antibody rates. Only two of 22 antibody-positive children were CFA positive.

### Molecular xenomonitoring results from surveys performed in 2015.

Four thousand mosquitoes in 200 pools (20 mosquitoes per pool) collected in the Balapitiya PHI area were tested for *W. bancrofti* DNA by qPCR (Table 2). Filarial DNA was detected in mosquitoes from 49 of 50 trap sites (Figure 2). A very high percentage of mosquito pools (65%) contained filarial DNA, and the upper CI for filarial DNA prevalence in mosquitoes was much higher than the target of < 1%. These MX results, together with high rates of antigenemia and anti-filarial antibody in primary school children, strongly suggest that LF transmission is ongoing in the study area.

**Table 2:** Filarial DNA prevalence in *Culex quinquefasciatus* in Public Health Midwife (PHM) areas within the Balapitiya Public Health Inspector that were tested in 2015 and in 2016

	MX in 2015					MX in 2016				
PHM	Number of HH* trap sites	Number of mosquitoes tested	Number of pools tested	Number (%) of positive pools	Filarial DNA prevalence in mosquitoes % (95% CI)	Number of HH trap sites	Number of mosquitoes tested	Number of pools tested	Number (%) of positive pools	Filarial DNA prevalence in mosquitoes % (95% CI)
Galmangoda	10	800	40	25 (62.5)	4.8 (2.9–7.3)	15	750	30	19 (63)	3.9 (2.2–6.4)
Brahmanawathugoda-South	10	800	40	29 (72.5)	6.2 (3.9–9.4)	15	750	30	13 (43)	2.2 (1.1–3.9)
Balapitiya	10	800	40	29 (72.5)	6.2 (3.9–9.4)	15	750	30	19 (63)	3.9 (2.2–6.4)
Randombe	10	800	40	26 (65)	5.1 (3.2–7.8)	15	750	30	13 (43)	2.2 (1.1–3.9)
Brahmanawathugoda-North	10	800	40	22 (55)	3.9 (2.3–6.1)	15	750	30	16 (53)	3.0 (1.6–5.0)
Total	50	4,000	200	131 (65.5)	5.2 (4.2–6.3)	75	3,750	150	80 (53)†	3.0 (2.3–3.8)†

CI = confidence interval; PHM = public health midwife.

* HH = household. Microfilaremia (Mf) surveys by the Anti-Filariasis Campaign in 2013 found Mf prevalences of 3.4%, 0.8%, 4.4%, 1.7% and 0.9% Mf prevalence in these PHM areas, respectively. Molecular xenomonitoring (MX) detected high percentages of mosquito pools positive for filarial DNA (mean 39%, range 20–60%) for filarial DNA in three Balapitiya PHM areas in 2014 surveys.^15^ Anti-Filariasis Campaign provided mass drug administration in Balapitiya in 2014 and in 2015.

† The decline in the percentages of mosquito pools positive for filarial DNA (combined data from five PHM areas) between 2015 and 2016 was not statistically significant. However, the decrease in estimated filarial DNA prevalence in mosquitoes between 2015 and 2016 was significant.

### Molecular xenomonitoring results from surveys performed in 2016.

Repeat MX surveys were conducted in the Balapitiya PHI area in 2016, approximately 6 months after the second of two rounds of supplemental MDA was provided by the MOH and after the MX survey conducted in 2015. Three thousand seven hundred and fifty mosquitoes in 150 pools (25 mosquitoes per pool) from 75 HH trap locations were tested by qPCR. Many pools were positive for filarial DNA (Table 2). Although the filarial DNA prevalence (maximum likelihood estimate) in mosquitoes was significantly lower in 2016 than in 2015, prevalence and the upper confidence interval values remained much higher than the 0.25 and 1% targets in all PHMs tested (Table 2). The difference in the percentage of mosquito pools positive for filarial DNA between the 2015 and 2016 surveys was not significant. These results show that two additional rounds of MDA were not sufficient to reduce filarial DNA rates in mosquitoes to low levels in Balapitiya.

**Figure 3.**
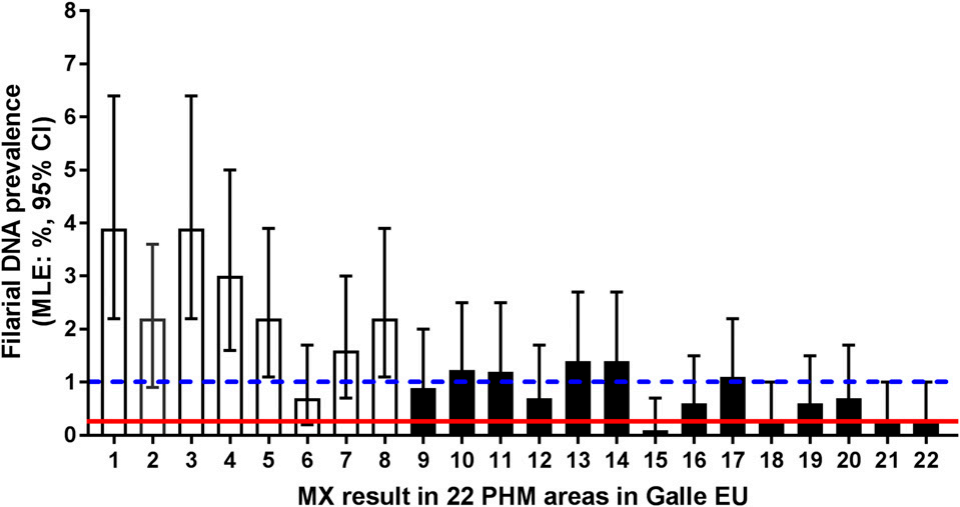
Reassessment of *Wuchereria bancrofti* in *Culex quinquefasciatus* in 2016 by molecular xenomonitoring (MX). The figure shows filarial DNA prevalence (MLE with 95% confidence intervals [CIs]) for mosquitoes collected in 22 public health midwife areas (PHMs) in the coastal Galle evaluation unit (EU). Public Health Midwife areas 1–8 (clear bars) are in the Balapitiya Ministry of Health area; PHM areas 9–22 (shaded bars) were additional sentinel sites in the Galle coastal evaluation unit that had high infection parameters in prior surveys. Red solid and blue dotted horizontal lines represent a filarial DNA prevalence (maximum likelihood estimate) of 0.25% and the 1% upper CI limit for the estimate that are provisional targets for lymphatic filariasis elimination, respectively. This figure appears in color at www.ajtmh.org.

Molecular xenomonitoring results from 2016 for 22 PHM areas in the Galle coastal EU are summarized in Figure 3. Approximately 660 pools were collected and tested, and these included 240 pools from the Balapitiya MOH area that were in three different PHI areas (i.e., not limited to the Balapitiya PHI area mentioned in the prior paragraph) (see Supplemental Table 1). Overall, 179 of 660 (27%) pools were positive for filarial DNA and a maximum likelihood estimate (MLE) of 1.26% (95% CI: 1.0–1.5%), and these prevalences were above the provisional target rate (an upper CI for the maximum likelihood rate of filarial DNA in mosquitoes of 1%). One hundred and seven of 240 pools (45%, range 16–63%) collected in the Balapitiya MOH area were positive for filarial DNA. The filarial DNA prevalence in these mosquitoes exceeded the provisional MX target in 18 of 22 (82%) PHMs tested (Figure 3, Supplemental Table 1). The pool screen results for MLEs, percentage of pools positive for filarial DNA, and an average number of trap locations positive for filarial DNA in mosquitoes are shown in Supplemental Table 1.

These results show that filarial DNA was still widespread in mosquitoes in the coastal Galle EU following two supplementary rounds of MDA provided in 2014 and 2015 and that the problem was not limited to the Balapitiya PHI or to the Balapitiya MOH area. They also suggest that mosquito monitoring may be preferable to Mf testing in humans for rapidly assessing the impact of interventions in areas with low residual infection rates in post-MDA settings. That is because MX signals for persisting LF were much stronger than those based on CFA or Mf survey results. Finally, these results suggest that additional rounds of routine MDA may not be sufficient to clear residual infections or interrupt transmission of LF in coastal Galle and that other interventions should be considered. These could include enhanced MDA with improved social mobilization and directly observed treatment, use of a new triple-drug MDA regimen that has recently been endorsed by the WHO,^22^ and new drug distribution activities designed to reach adult males outside of their homes.

### Additional community Mf survey results.

The AFC’s night blood survey in 2016 tested 16,927 people from the age of 2 years in areas that included both Balapitiya and the adjacent area of Wathugedara 6 months after the MDA that was provided in 2015. Ninety-seven blood smears were positive for *W. bancrofti* microfilariae (prevalence 0.6%, CI: 0.47–0.71%). Microfilaremia prevalence was marginally higher in Balapitiya (85 of 13,927; 0.6%, CI: 0.59–0.75%) than in Wathugedara (12 of 2,841; 0.42%, CI: 0.24–0.74%). In 2016 the overall Mf prevalence in Galle EU by night blood smear was 0.4% (155 of 41,928). One hundred and forty-two of 155 (92%) Mf-positive cases were reported from Balapitiya MOH area.

We previously reported that mosquito infections were high in many areas in the Galle coastal EU that had a surveyed Mf rate of 0.2% in 2013.^15^ Our new Mf data and MX results show again that mosquito infection rates can be very high in focal areas within EU with overall surveyed Mf rates of less than 1% in that EU. Thus, routine Mf testing is insensitive for detecting persistent LF in post-MDA situations with low residual infection rates, and the surveys are notoriously difficult to perform well. Night blood Mf surveys may underestimate true prevalence rates because of technical problems with the smears or to nonparticipation of Mf carriers in surveys. Mosquitoes do not require participant consent when they sample blood. Our MX results suggest that Mf carriers who were noncompliant with MDA in the past and not sampled in the government’s night blood surveys serve as a persistent reservoir of infection that contributes to continued LF transmission in hotspots such as Balapitiya in Sri Lanka.

### Spatial distribution of filarial infections in humans and mosquitoes.

**Figure 4.**
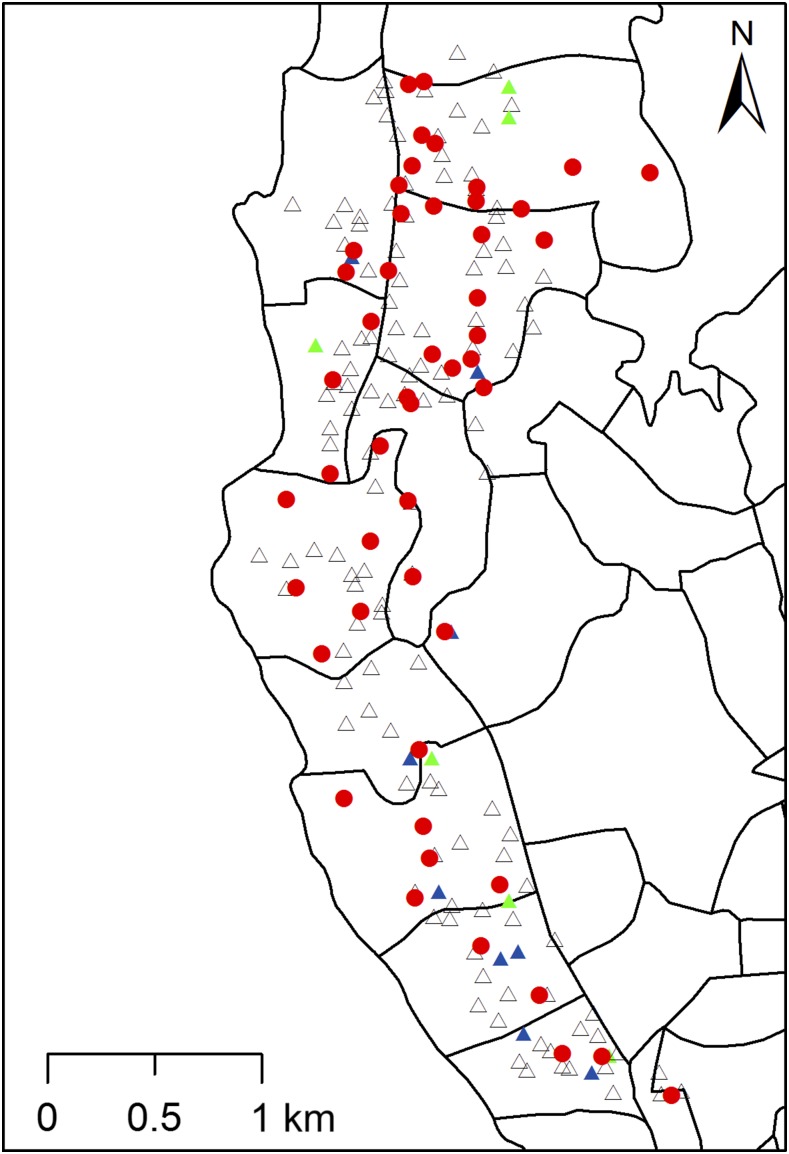
Distribution of households (HHs) and mosquito collection sites tested for filarial infection in the Balapitiya Public Health Inspector area. Blue and green triangles indicate HHs where at least one resident had a positive filarial antigen test or microfilaremia, respectively. Households with no positive filarial antigen test are shown in open black triangles. Trap locations with one or more mosquito pools positive for filarial DNA are shown with red circles. Filarial DNA was detected in mosquitoes from 49 of 50 trap sites. Thus, filarial infection was widespread in the study area at the time of the survey. This figure appears in color at www.ajtmh.org.

Figure 4 shows locations of HHs with one or more residents that tested positive for CFA and Mf and of mosquito trap sites that yielded pools that were positive for filarial DNA. Circulating filarial antigenemia and Mf were detected in residents of 15 of 140 (11%) and in six of 140 (4%) houses surveyed along the length of the PHI; filarial DNA was detected in mosquitoes collected in 49 of 50 traps placed in the study area.

The median distance from CFA-positive HHs (*N* = 15) to the closest positive mosquito trap was 88.9 meters (range 28–397 m), and the median distance from CFA-negative HHs (*N* = 124) to the closest positive trap was 128 m (range 8–401 m). This difference is not statistically significant (Wilcoxon rank sum test [*P* = 0.4]). It is possible that the number of positive houses in the study was too small to detect a spatial relationship between human infections and positive mosquito-trapping locations. However, prior studies by our group and others have also failed to document this type of spatial relationship even when infection rates were higher.^14,23–25^ This suggests that *Culex* mosquitoes range widely in Sri Lanka during the interval between blood meals and oviposition. It is also possible that a significant amount of mosquito feeding occurs outside of the home environment.

### Additional discussion and conclusions.

This study has provided useful information on the assessment of persistent filarial infections and probable ongoing transmission in Balapitiya PHI and in other areas in the coastal Galle district EU following multiple rounds of MDA. Two prior studies have shown persistence of *W. bancrofti* in populations approximately 6–9 years following five rounds of MDA in several EUs in Sri Lanka including the high-risk coastal Galle EU. This persistence is incongruous with the fact that all formerly endemic EUs in the country including coastal Galle EU easily passed TAS in 2013. Those TAS results (together with other information) led WHO to validate that Sri Lanka had eliminated LF as a public health problem in 2016.

A post-MDA surveillance study in American Samoa (where LF is transmitted by *Aedes polynesiensis*) found residual infections with ongoing transmission in some hotspots^26^ after the EU had passed TAS. Our results confirm that passing school-based TAS at the EU level does not prove that LF has been eliminated from the entire EU. This study has provided solid field data to support model-based predictions in a recent publication that addressed heterogeneities in individual infection distributions in communities that have completed MDA and exposure heterogeneities that can lead to LF hotspots in areas that pass TAS in large EUs.^12,27^ Additional research is needed to improve post-MDA surveillance methods and to develop and test methods for hotspot removal. The Sri Lanka AFC has started to perform additional surveillance in humans and in mosquitoes to search for additional foci with persistent LF in the Galle coastal EU.

Results from this study demonstrate the value of comprehensive surveys including MX for evaluating potential hotspots in EUs that have passed school-based TAS. The Balapitiya PHI meets our provisional definition for a LF hotspot because it is an area smaller than an EU that has Mf prevalence with a 95% CI of ≥ 1% in adults despite five or more rounds of effective MDA.

Antigen prevalence data from this study and in other recent publications show that adult males account for most persistent filarial infections in Sri Lanka at this time,^9,14^ and this is also true in other regions that have received multiple rounds of MDA (authors’ unpublished data). Therefore, wider application of MX and shifting the focus of surveillance from children (which detects more recent infections) to adults (to assess the persisting reservoir of infection) might improve post-MDA surveillance and hotspot detection. In a similar way, new tools including an improved treatment regimen and targeted MDA to increase compliance among high-risk groups may sometimes be required to remove hotspots and traverse the difficult the last mile to LF elimination.

## Supplementary Material

Supplemental table
