# Supplementary material for: Comprehensive Assessment of a Hotspot with Persistent Bancroftian Filariasis in Coastal Sri Lanka
Source: Am J Trop Med Hyg. 2018 Jul 16;99(3):735–42. doi: 10.4269/ajtmh.18-0169 (PMC6169179; doi:10.4269/ajtmh.18-0169)
Supplement: Supplementary file 1 [file tpmd180169.SD1.doc]

Supplementary Table 1. Summary of 2016 molecular xenomonitoring results from 22 Public Heath Midwife (PHM) areas in the coastal Galle evaluation unit.

| MOH Area | PHI Area | PHM Area | ^a^, Number of Pools tested by qPCR | Number of Pools positive for filarial DNA (%) and *MLE [%, 95% CI]* | Number of Traps positive for filarial DNA in mosquitoes (%) |
| --- | --- | --- | --- | --- | --- |
| Balapitiya | Balapitiya | Balapitiya | 30 | 19 (63.3) | 13 (86.7) |
|  | Balapitiya | Randombe | 30 | 13 (43.3) | 10 (66.6) |
|  | Balapitiya | Brahmanawaththa-North | 30 | 16 (53.3) | 12 (80.0) |
|  | Balapitiya | Brahmanawaththa-South | 30 | 13 (43.3) | 9 (60.0) |
|  | Balapitiya | Galmangoda | 30 | 19 (63.3) | 11 (73.3) |
|  | Wathugedara | Wathugedara-01 | 30 | 5 (16.7) | 4 (26.7) |
|  | Wathugedara | Paragahathota | 30 | 12 (40.0) | 10 (66.7) |
|  | Ahungalla | Piygama | 30 | 10 (33.3) | 8 (53.3) |
|  |  | Sub total | 240 | 107 (44.6) [*2.3%, 1.8-2.8*] | 77 (64.2) |
| Habaraduwa | Ahangama | Danduhela | 30 | 7 (23.3) | 7 (46.7) |
|  | Unawatuna | Thalpe-01 | 30 | 4 (13.3) | 4 (26.7) |
|  | Kathaluwa | Palassa | 30 | 1 (3.3) | 1 (16.7) |
|  |  | Sub total | 90 | 12 (13.3) [*0.57%, 0.2-1.0*] | 12 (26.7) |
| Akmeemana | Walahanduwa | Bataduwa | 30 | 9 (30.0) | 7 (46.7) |
|  | Walahanduwa | Ethiligoda | 30 | 2 (6.7) | 2 (13.3) |
|  | Wanchawala | Kalahe | 30 | 5 (16.7) | 3 (20.0) |
|  |  | Sub total | 90 | 16 (17.8) [*0.78%, 0.4-1.3*] | 12 (26.7) |
| Galle M.C. | Richmand Hill | Madawalamulla -North | 30 | 8 (26.7) | 4 (26.7) |
|  | Dangedara | Ethiligoda | 30 | 6 (20.0) | 6 (40.0) |
|  | Magalla | Magalla | 30 | 8 (26.7) | 7 (46.7) |
|  |  | Sub total | 90 | 22 (24.4) [*1.11%, 0.4-1.3*] | 17 (37.8) |
| Hikkaduwa | Hikkaduwa | Wavulegoda | 30 | 5 (16.7) | 5 (33.3) |
|  | Hikkaduwa | Nalagasdeniya | 30 | 4 (13.3) | 4 (26.7) |
|  | Madampagama | Wenamulla | 30 | 2 (6.7) | 2 (13.3) |
|  |  | Sub total | 90 | 11 (12.2) [*0.52%, 0.2-0.9*] | 11 (24.4) |
| Rathgama | Boossa | Boossa | 30 | 2 (6.7) | 1 (6.7) |
|  |  | Sub total | 30 | 2 (6.7) [*0.28%, 0.03-0.9*] | 1 (6.7) |
| Ambalangoda | Ambalangoda | Patabandimulla | 30 | 9 (30.0) | 7 (46.7) |
|  |  | Sub total | 30 | 9 (30.0) [*1.42%, 0.6-2.7*] | 7 (46.7) |
|  |  | Total | 660 | 179 (27.1) [*1.26%, 1.0-1.5*] | 137 (41.5) |

^a^, 30 *Cx. quinquefasciatus* pools containing 25 mosquitoes per pool were collected from 15 trap locations in each PHM area and tested for filarial DNA.
